# In Silico Analysis of the Missense Variants of Uncertain Significance of *CTNNB1* Gene Reported in GnomAD Database

**DOI:** 10.3390/genes15080972

**Published:** 2024-07-24

**Authors:** Arturo Caballero-Avendaño, Melva Gutiérrez-Angulo, María de la Luz Ayala-Madrigal, José Miguel Moreno-Ortiz, Anahí González-Mercado, Jorge Peregrina-Sandoval

**Affiliations:** 1 Doctorado en Genética Humana e Instituto de Genética Humana, Centro Universitario de Ciencias de la Salud, Guadalajara 44340, Mexico; arturo.caballero2313@alumnos.udg.mx (A.C.-A.); melva.gutierrez@academicos.udg.mx (M.G.-A.); luz.ayala@academicos.udg.mx (M.d.l.L.A.-M.); miguel.moreno@academicos.udg.mx (J.M.M.-O.); anahi.gonzalez@academicos.udg.mx (A.G.-M.); 2 Departamento de Ciencias de la Salud, Centro Universitario de los Altos, Tepatitlán de Morelos 47600, Mexico; 3 Instituto de Fisiología Celular del Departamento de Biología Celular y Molecular, Centro Universitario de Ciencias Biológicas y Agropecuarias, Zapopan 45200, Mexico

**Keywords:** in silico analysis, VUS, cancer, CTNNB1 gene, missense

## Abstract

*CTNNB1* pathogenic variants are related to the improper functioning of the WNT/β-catenin pathway, promoting the development of different types of cancer of somatic origin. Bioinformatics analyses of genetic variation are a great tool to understand the possible consequences of these variants on protein structure and function and their probable implication in pathologies. The objective of this study is to describe the impact of the missense variants of uncertain significance (VUS) of the *CTNNB1* gene on structure and function of the β-catenin protein. The *CTNNB1* variants were obtained from the GnomAD v2.1.1 database; subsequently, a bioinformatic analysis was performed using the VarSome, UCSC Genome Browser, UniProt, the Kinase Library database, and DynaMut2 platforms to evaluate clinical significance, gene conservation, consensus sites for post-translational modifications, and the dynamics and stability of proteins. The GnomAD v2.1.1 database included 826 variants of the *CTNNB1* gene, of which 385 were in exons and exon/intron boundaries. Among these variants, 214 were identified as missense, of which 146 were classified as VUS. Notably, 12 variants were in proximity to consensus sites for post-translational modifications (PTMs). The in silico analysis showed a slight tendency towards probably pathogenic for c.59C>T (p.Ala20Val) and c.983T>C (p.Met328Thr) missense VUS. These findings provide possible functional implications of these variants in some types of cancer.

## 1. Introduction

The *CTNNB1* gene is located at 3p22.1 and contains 15 exons (14 coding) (ENST00000349496.11) [1]. This gene encodes the β-catenin protein, which is essential for maintaining cell adhesion and regulating transcription activated by the WNT signaling pathway [2]. Some *CTNNB1* variants may lead to dysregulation of the WNT/β-catenin pathway, promoting the development of various neoplasms such as colorectal, melanoma, medulloblastoma, cervical–uterine, bladder, prostate, stomach, and liver cancer. Additionally, these variants might be implicated in neurodevelopmental disorders, manifesting as behavioral abnormalities, anxiety, impaired motor function, and cognitive deficits [2,3,4].

The Genome Aggregation Database (GnomAD v2.1.1) includes genomic data from unrelated individuals (64,754 females and 76,702 males) without a medical or family history of severe pediatric disease. These data were collected from various populations: non-Finnish European (n = 64,603), Latino (n = 17,720), South Asian (n = 15,308), Finnish (n = 12,562), African American (n = 12,487), East Asian (n = 9977), Ashkenazi Jewish (n = 5185), and other (n = 3614); ancestry was determined using principal component analysis and random forest [5]. The gene-specific missense variants with unknown clinical significance reported in this database can be analyzed through bioinformatic tools to determine their effects on protein stability, consensus sites for post-translational modifications (PTMs), protein interactions, or catalytic sites [6].

The aim of this study is to describe the possible impact of missense variants of uncertain significance (VUS) of the *CTNNB1* gene, which are reported in the GnomAD v2.1.1 database, on the structure and function of the β-catenin protein.

## 2. Materials and Methods

### 2.1. Selection of Missense VUS 

**Figure 1:**
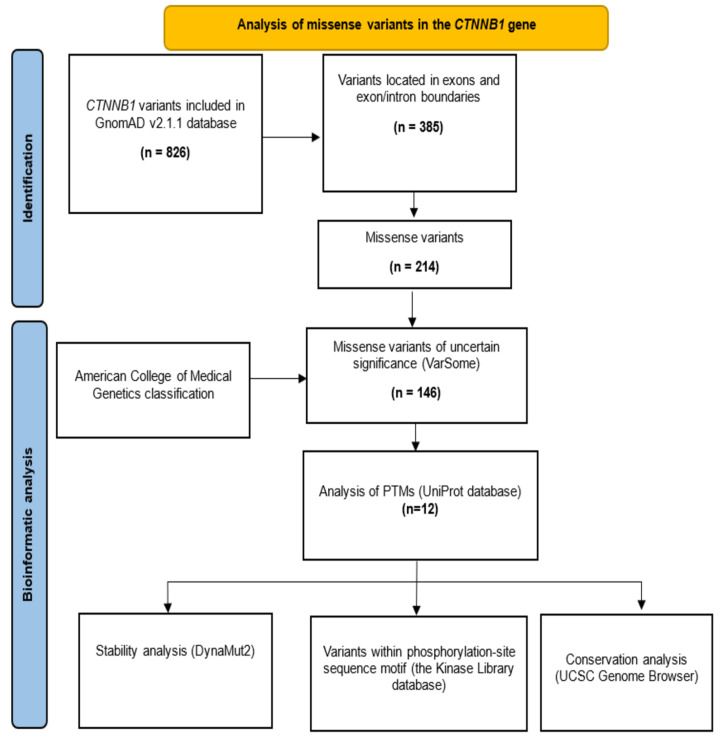
Methodological diagram for the analysis of missense VUS of the *CTNNB1* gene. PTMs: post-translational modifications. UCSC: University of California Santa Cruz.

The missense variants of the *CTNNB1* gene were obtained from the GnomAD v2.1.1 database (https://gnomad.broadinstitute.org/, accessed on 1 March 2023) [5]. The guidelines described by the Human Genome Variation Society (HGVS) were followed using the sequence NM_001904.4 as a reference. The germline classification was revised in the free VarSome v11.17.0 database [7]. Those classified as VUS were selected for in silico analysis (Figure 1).

### 2.2. Impact of Missense VUS on Phosphorylation Sites

The relationship between missense VUS and consensus sites for PTMs was examined using the UniProt (https://www.uniprot.org/, accessed on 28 September 2023, ID: P35222) and PhosphoSitePlus databases (https://www.phosphosite.org/homeAction.action, accessed on 2 October 2023) [8,9]. Additionally, phosphorylation site sequence motifs were consulted in the the Kinase Library database (https://kinase-library.phosphosite.org/site, accessed on 17 April 2024) [6]. The scoring system used is as follows: Log_2_ score = 0: neutral; Log_2_ score > 0: favorable; and Log_2_ score < 0: unfavorable [6].

### 2.3. Conservation Analysis of Affected Residues

The level of sequence conservation was determined using the PhyloP metrics from the UCSC Genome Browser database (https://genome.ucsc.edu/, accessed on 23 October 2023). These analyses are used to calculate the conservation scores based on a phylo-HMM (hidden Markov model). Sites predicted to be conserved are assigned positive scores (ranging between 0 and 1), while sites predicted to be fast evolving are assigned negative scores [10]. 

### 2.4. Stability Analysis of Missense VUS

Finally, an in silico analysis of the β-catenin protein (ID: P35222) was conducted using the DynaMut2 platform (https://biosig.lab.uq.edu.au/dynamut2/, accessed on 22 April 2024) to determinate the dynamics and stability of the protein as a result of changes in vibrational entropy [11]. The structure used for analysis was AF-P35222-F1, which was predicted by AlphaFold [12]. The following cutoff points were used for the interpretation of the results: ΔΔGStability > 0.0 kcal/mol is destabilizing, and ΔΔGStability < 0.0 kcal/mol is stabilizing [11].

## 3. Results

### 3.1. Analysis of Variants of the CTNNB1 Gene

**Figure 2:**
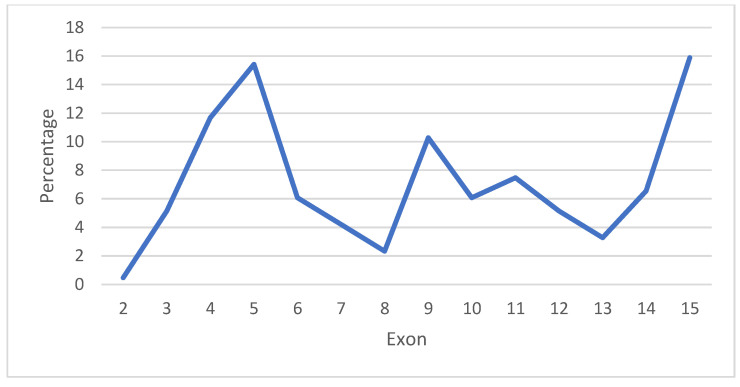
Distribution of 214 missense variants of *CTNNB1* gene obtained from GnomAD v2.1.1 database.

The GnomAD v2.1.1 database included 826 variants of the *CTNNB1* gene, with 385 located in exons and exon/intron boundaries. Among these, 214 were missense, 167 were synonyms, one was an in-frame deletion, one was a start-lost variant, and two were splice acceptor variants (Table 1). Neither nonsense nor frameshift variants were found in the population reported by GnomAD v2.1.1. The missense variants were primarily located in exon 15 (15.9%), exon 5 (15.4%), exon 4 (11.7%), and exon 9 (10.3%) (Figure 2). According to the VarSome database [7], the missense variants were mainly classified as VUS (68%), and only 1% are likely pathogenic (Table 2). 

Supplementary Table S1 includes the 12 variants that were analyzed and details the rsID, HGVS nomenclature (gene and protein), PTM sites, affinity score for kinases, conservation, protein stability, and allele frequency.

### 3.2. In Silico Analysis of Phosphorylation Motif Sequence

The UniProt database contains 30 sites for PTMs in the β-catenin protein, and 12 missense VUS were reported within these consensus sites. However, the enzyme implicated in the modification was reported for only six of these sites (Supplementary Table S1). Additionally, only the consensus sequences for GSK3B and CDK5 were obtained from the Kinase Library database [6].

**Figure 3:**
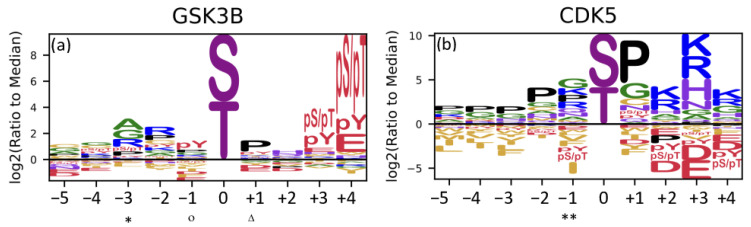
Sequence motif for GSK3B and CDK5. (**a**) Sequence for GSK3B. The −3 (*), −1 (°), and +1 (Δ) positions are affected by c.59C>T (p.Ala20Val), c.84G>T (p.Gln28His), and c.125C>T (p.Thr42Ile), respectively. (**b**) Sequence for CDK5. The −1 (**) position is affected by the variant c.569G>A (p.Arg190His). (Image obtained from Johnson et al., 2022 [6]; adapted using https://app.biorender.com/biorender-templates, accessed on 12 February 2024).

The variants c.59C>T (p.Ala20Val), c.84G>T (p.Gln28His), and c.125C>T (p.Thr42Ile) were located in the recognition site for GSK3B at the −3, −1, and +1 positions, respectively. However, only the c.59C>T (p.Ala20Val) variant showed a decreased affinity (the log_2_ score was 0.818 and –0.034 for the reference and alternative alleles, respectively) (Figure 3a). The variant c.569G>A (p.Arg190His) was located in the CDK5 recognition site at the −1 position, but no impact on the affinity was found (the log_2_ score was −0.289 and 0.385 for the reference and alternative alleles, respectively) (Figure 3b).

**Figure 4:**
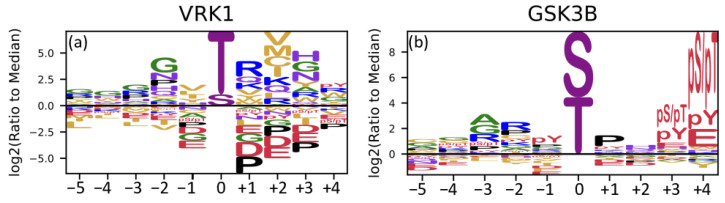
Sequence motif for VRK1 (**a**) and GSK3B (**b**). The 0 position is the new site generated by the variants c.983T>C (p.Met328Thr) and c.152A>G (p.Asn51Ser). (Image obtained from Johnson et al., 2022 [6]; adapted using https://app.biorender.com/biorender-templates, accessed on 12 February 2024).

Regarding the variant c.983T>C (p.Met328Thr), the presence of the threonine residue generates a new phosphorylation recognition site for the kinase VRK1 (Figure 4a). Similarly, for the variant c.152A>G (p.Asn51Ser), the presence of the serine residue is predicted to generate a phosphorylation recognition site for GSK3B (Figure 4b).

### 3.3. Impact on and Changes in the Protein Structure Analysis

**Figure 5:**
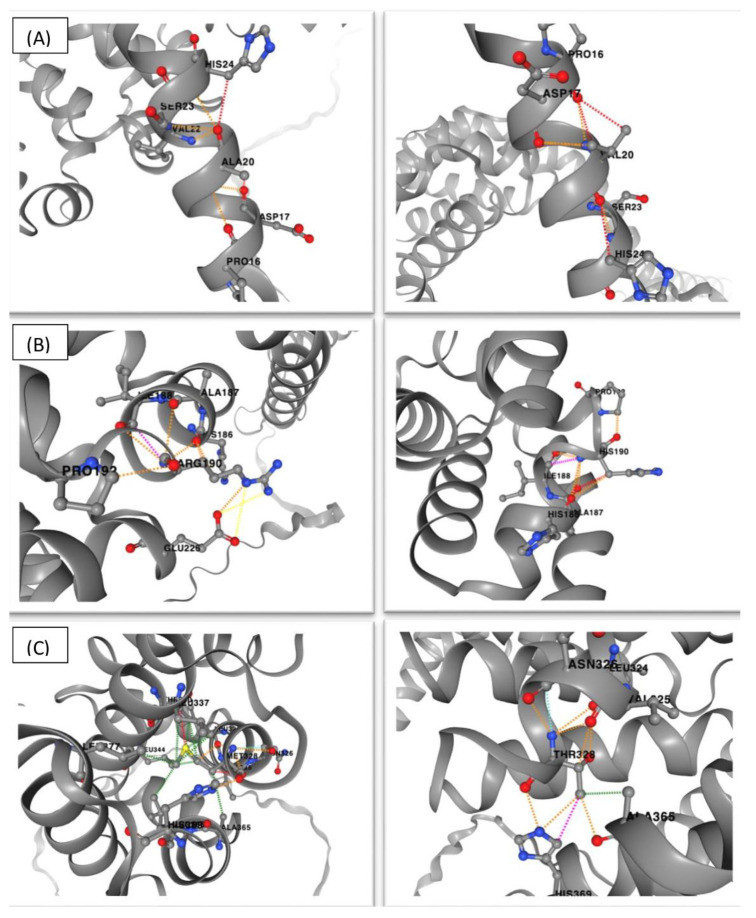
DynaMut2 results for (**A**) p.Ala20Val, (**B**) p.Arg190His, and (**C**) p.Met328Thr variants. The changes in amino acid interactions are shown with dotted lines in wild-type (**left**) and mutated forms (**right**) of β-catenin.

The stability analysis using the DynaMut2 platform showed a destabilizing impact for 11 missense VUS (Supplementary Table S1). Different amino acid interactions were observed in the wild-type protein compared to the mutated protein containing the new amino acid that was introduced by the β-catenin variant, as illustrated in Figure 5, which shows the three variants that had the greatest impact on stability (p.Ala20Val, p.Arg190His, and p.Met328Thr).

### 3.4. Conservation Analysis

Additionally, a conservation analysis showed that 67% (8 of 12) of the *CTNNB1* variants were highly conserved, 25% were moderately conserved, and 8% were poorly conserved (Supplementary Table S1).

## 4. Discussion

The VUS have significant relevance due to the challenges in genetic counseling and clinical management [13]. An in silico analysis of missense VUS can predict potential effects on protein structure and/or function and may contribute to the reclassification of these variants. 

The analysis of *CTNNB1* variants in the GnomAD v2.1.1. database showed 214 missense variants distributed mainly in exons 15, 5, 4, and 9. The analysis showed that 146 variants were classified as VUS, but only 12 were related to consensus sites for PTMs. The amino acid changes in the missense variants can affect the phosphorylation site motif, thereby potentially altering protein function. In this regard, the variant c.59C>T (p.Ala20Val) showed a decreased affinity for GSK3B. This kinase phosphorylates the residue Ser23, and it is hypothesized to be involved in abnormal cell growth and the ubiquitination process [14]. Therefore, variants within the recognition site of GSK3B could affect its phosphorylation and alter the normal function of the protein; since β-catenin is involved in cell–cell adhesion and transcriptional regulation, this variant could promote tumor development. The variant p.Ala20Val has been reported in esophageal, large intestine, thyroid, and skin carcinomas [15]. In a study conducted by Vasovcak et al. in 2011 [16], which involved 103 tumors from 102 colorectal cancer patients, two mutations in *CTNNB1* were identified: p.Ser45Phe, a well-known pathogenic variant, and p.Ala20Ser, which the authors concluded was likely not associated with the disease. 

The variants may also introduce new sites for PTMs. According to the analysis conducted using the Kinase library database, the variants c.152A>G (p.Asn51Ser) and c.983T>C (p.Met328Thr) created new sites for phosphorylation mediated by GSK3B and VRK1, respectively. Both kinases have been implicated in the regulation of transcription factors and cell cycle progression. GSK3B is well known for its role in β-catenin regulation [17]; however, VRK1 could be a novel kinase involved in the regulation of this protein. VRK1 is the most abundant nuclear kinase, is overexpressed in many types of cancer, and is involved in cell proliferation [18]. Phosphorylation is one of the most important reversible PTMs, and serine, tyrosine, and threonine are the most studied residues; however, phosphorylation also occurs in other non-canonical amino acids such as histidine, aspartate, glutamate, lysine, arginine, and cysteine. This PTM can affect protein folding, function, stability, interaction, and localization, and it has been implicated in several cellular biological activities, including signal transduction, translation, transcription, cell division, DNA repair, and apoptosis [19,20]. In this regard, the generation of new phosphorylation sites in the protein could add new cellular functions that could enhance its effect on cancer development or have a negative effect on tumor progression. The variant p.Asn51Ser has been described in a male patient with sinonasal lymphoma of the natural killer/T-cell subtype [21]. Additionally, the same residue (Asn51) was affected in two males with liver carcinoma: p.Asn51Thr, reported in the COSMIC database (COSMIC mutation ID: COSV62698188), and p.Asn51Lys, reported in the cBioPortal database [15,22]. However, p.Met328Thr has not been reported in cancer [15,22], though the residue Met328 was substituted by isoleucine in a female patient with non-small cell lung cancer [22]. Notably, an analysis using Polyphen2 [23] indicated a possible damaging effect exclusively for p.Met328Thr (score 0.759). However, for both variants (p.Asn51Ser and p.Met328Thr), the oncogenic and biological effects remain unclear.

Stability analysis was conducted on 12 variants and revealed a destabilizing impact in 92% of them, which could have negative consequences regarding the biological activity of the protein. However, it is important to highlight that these alterations can be harmful or even beneficial depending on their nature and location. The variants c.59C>T (p.Ala20Val), c.84G>T (p.Gln28His), c.125C>T (p.Thr42Ile), c.152A>G (p.Asn51Ser), and c.412A>G (p.Asn138Asp), which are located in the N-terminal domain (residues 1–150), are implicated in degradation; c.983T>C (p.Met328Thr) is located in armadillo repeat 5 (residues 319–360), a critical region for APC binding and cell–cell adhesion; c.1660G>T (p.Gly554Cys) is positioned in armadillo repeat 10 (residues 531–571) and is important for protein stability and cell–cell adhesion; c.2003A>G (p.Gln668Arg) and c.2042C>T (p.Ser681Phe) are situated in the helix C domain (residues 667–683), which is critical for interaction with transcriptional coactivators; and c.2149C>T (p.Arg717Cys) is located in the C-terminal domain (residues 684–781), which is also involved in transcriptional regulation related to the WNT signaling pathway [24,25]. Therefore, variants that affect the protein stability could impact degradation, cell adhesion, or transcriptional activity, which are the main functions of β-catenin. Out of the 12 variants, 11 were found to destabilize the protein, with only 5 reported in cancer (p.Ala20Val, p.Thr42Ile, p.Arg190His, p.Gly554Cys, and p.Arg717Cys) [15,22]. Additionally, evidence has shown that the Gln28 residue was changed to Glu or Arg in lung and liver carcinomas, respectively, while Asn138 was replaced by His or Lys in colorectal and prostate cancer, respectively [15,22].

Although 92% of the variants had a destabilizing impact on β-protein, p.Ala20Val, p.Arg190His, and p.Met328Thr showed higher scores regarding protein stability. The first variant was located in the N-terminal domain, and the others in ARM repeats; therefore, degradation and cell adhesion could be affected, and this variant could contribute to tumor development. Notably, the variant p.Arg190His has been reported in colorectal, lung, and prostate cancer [15,22].

In addition, a conservation analysis showed that the variants were located in highly conserved regions; therefore, a change in these amino acids could affect protein function.

The GnomAD database v2.1.1. includes 146 missense VUS and only 12 were analyzed in this study. Consequently, the main limitation was the small number of variants analyzed due a limited availability of sequence motifs for PTMs since more than one million of the PTMs have been estimated [26]. It would be interesting to analyze all the variants to determine if they are implicated in consensus sites for the main PTMs and to predict the effects on the β-catenin protein. Moreover, further analyses could be conducted on variants located in regulatory regions to predict their effects on protein levels.

## 5. Conclusions

Based on this in silico analysis, the c.59C>T (p.Ala20Val) and c.983T>C (p.Met328Thr) variants had an impact on protein stability, and a phosphorylation motif was lost and created, respectively, suggesting a slight tendency towards probable pathogenicity. Although the population analyzed in the GnomAD database was apparently healthy, these variants could potentially contribute to tumor development. Moreover, it is important to highlight that sporadic cancer is produced by multiple mutations, and low penetrance variants like these may increase cancer risk. Furthermore, analyses of VUS using bioinformatic tools are essential to predict functional implications due to changes in protein structure or loss/gain of PTMs that may influence biological functions and contribute to diseases, although functional assays are required to validate the impact on the β-catenin protein.

## Figures and Tables

**Table 1:** Distribution of the 385 variants located in exons and exon/intron boundaries in *CTNNB1* gene.

*CTNNB1* Variant Type	N (%)
Missense	214 (56)
Synonymous	167 (43)
Others	4 (1)

**Table 2:** ACMG classification of the 214 missense variants of *CTNNB1* gene.

ACMG Classification	N (%)
Likely pathogenic	3 (1)
VUS	146 (68)
Likely benign	54 (25)
Benign	11 (5)

ACMG: American College of Medical Genetics. VUS: variant of uncertain significance.

## Data Availability

Data is contained within the article and Supplementary Material.

## Supplementary Materials

The following supporting information can be downloaded at https://www.mdpi.com/article/10.3390/genes15080972/s1: Table S1: In silico analysis of missense VUS of the *CTNNB1* gene reported in the GnomAD v2.1.1 database.

## References

[1] Sayers E.W., Beck J., Brister J.R., Bolton E.E., Canese K., Comeau D.C., Funk K., Ketter A., Kim S., Kimchi A. (2020). Database resources of the National Center for Biotechnology Information. Nucleic Acids Res..

[2] Kim S., Jeong S. (2019). Mutation hotspots in the β-catenin gene: Lessons from the human cancer genome databases. Mol. Cells.

[3] Chang M.T., Asthana S., Gao S.P., Lee B.H., Chapman J.S., Kandoth C., Gao J.J., Socci N.D., Solit D.B., Olshen A.B. (2016). Identifying recurrent mutations in cancer reveals widespread lineage diversity and mutational specificity. Nat. Biotechnol..

[4] Zhuang W., Ye T., Wang W., Song W., Tan T. (2023). CTNNB1 in neurodevelopmental disorders. Front. Psychiatry.

[5] Karczewski K.J., Francioli L.C., Tiao G., Cummings B.B., Alföldi J., Wang Q., Collins R.L., Laricchia K.M., Ganna A., Birnbaum D.P. (2020). The mutational constraint spectrum quantified from variation in 141,456 humans. Nature.

[6] Johnson J.L., Yaron T.M., Huntsman E.M., Kerelsky A., Song J., Regev A., Lin T.Y., Liberatore K., Cizin D.M., Cohen B.M. (2023). An atlas of substrate specificities for the human serine/threonine kinome. Nature.

[7] Kopanos C., Tsiolkas V., Kouris A., Chapple C.E., Albarca Aguilera M., Meyer R., Massouras A. (2019). VarSome: The human genomic variant search engine. Bioinformatics.

[8] Bateman A., Martin M.J., Orchard S., Magrane M., Agivetova R., Ahmad S., Alpi E., Bowler-Barnett E.H., Britto R., Bursteinas B. (2021). UniProt: The universal protein knowledgebase in 2021. Nucleic Acids Res..

[9] Hornbeck P.V., Zhang B., Murray B., Kornhauser J.M., Latham V., Skrzypek E. (2015). PhosphoSitePlus, 2014: Mutations, PTMs and recalibrations. Nucleic Acids Res..

[10] Kent W.J., Sugnet C.W., Furey T.S., Roskin K.M., Pringle T.H., Zahler A.M., Haussler D. (2002). The Human Genome Browser at UCSC. Genome Res..

[11] Rodrigues C.H.M., Pires D.E.V., Ascher D.B. (2021). DynaMut2: Assessing changes in stability and flexibility upon single and multiple point missense mutations. Protein Sci..

[12] Jumper J., Evans R., Pritzel A., Green T., Figurnov M., Ronneberger O., Tunyasuvunakool K., Bates R., Žídek A., Potapenko A. (2021). Highly accurate protein structure prediction with AlphaFold. Nature.

[13] Corso G., Corso F., Bellerba F., Carneiro P., Seixas S., Cioffi A., La Vecchia C., Magnoni F., Bonanni B., Veronesi P. (2021). Geographical distribution of e-cadherin germline mutations in the context of diffuse gastric cancer: A systematic review. Cancers.

[14] Ha J.R., Hao L., Venkateswaran G., Huang Y.H., Garcia E., Persad S. (2014). β-Catenin is O-GlcNAc glycosylated at Serine 23: Implications for β-catenin’s subcellular localization and transactivator function. Exp. Cell Res..

[15] Tate J.G., Bamford S., Jubb H.C., Sondka Z., Beare D.M., Bindal N., Boutselakis H., Cole C.G., Creatore C., Dawson E. (2019). COSMIC: The Catalogue of Somatic Mutations in Cancer. Nucleic Acids Res..

[16] Vasovcak P., Pavlikova K., Sedlacek Z., Skapa P., Kouda M., Hoch J., Krepelova A. (2011). Molecular genetic analysis of 103 sporadic colorectal tumours in Czech patients. PLoS ONE.

[17] Stamos J.L., Weis W.I. (2013). The β-catenin destruction complex. Cold Spring Harb. Perspect. Biol..

[18] Campillo-Marcos I., García-González R., Navarro-Carrasco E., Lazo P.A. (2021). The human VRK1 chromatin kinase in cancer biology. Cancer Lett..

[19] Hardman G., Perkins S., Brownridge P.J., Clarke C.J., Byrne D.P., Campbell A.E., Kalyuzhnyy A., Myall A., Eyers P.A., Jones A.R. (2019). Strong anion exchange-mediated phosphoproteomics reveals extensive human non-canonical phosphorylation. EMBO J..

[20] Esmaili F., Pourmirzaei M., Ramazi S., Shojaeilangari S., Yavari E. (2023). A Review of Machine Learning and Algorithmic Methods for Protein Phosphorylation Site Prediction. Genom. Proteom. Bioinform..

[21] Kurniawan A.N., Hongyo T., Hardjolukito E.S., Ham M.F., Takakuwa T., Kodariah R., Hoshida Y., Nomura T., Aozasa K. (2006). Gene mutation analysis of sinonasal lymphomas in Indonesia. Oncol. Rep..

[22] de Bruijn I., Kundra R., Mastrogiacomo B., Tran T.N., Sikina L., Mazor T., Li X., Ochoa A., Zhao G., Lai B. (2023). Analysis and Visualization of Longitudinal Genomic and Clinical Data from the AACR Project GENIE Biopharma Collaborative in cBioPortal. Cancer Res..

[23] Adzhubei I., Jordan D.M., Sunyaev S.R. (2013). Predicting functional effect of human missense mutations using PolyPhen-2. Curr. Protoc. Hum. Genet..

[24] Van der Wal T., van Amerongen R. (2020). Walking the tight wire between cell adhesion and WNT signalling: A balancing act for β-catenin. Open Biol..

[25] Gao C., Wang Y., Broaddus R., Sun L., Xue F., Zhang W. (2017). Exon 3 mutations of *CTNNB1* drive tumorigenesis: A review. Oncotarget.

[26] Kitamura N., Galligan J.J. (2023). A global view of the human post-translational modification landscape. Biochem. J..

