# Supplementary material for: In Silico Analysis of the Missense Variants of Uncertain Significance of CTNNB1 Gene Reported in GnomAD Database
Source: Genes (Basel). 2024 Jul 24;15(8):972. doi: 10.3390/genes15080972 (PMC11353749; doi:10.3390/genes15080972)
Supplement: Supplementary file 1 [file genes-15-00972-s001.zip › genes-3114390-supplementary.pdf]

Table S1: In silico analysis of missense VUS of the *CTNNB1* gene reported in the GnomAD v2.1.1 database.

rsID	HGVS nomenclature (Gene)	HGVS nomenclature (Protein)	Phosphorylation site (UniProt/Phosphosite Plus)	Affinity log_2 score (The Kinase Library database)	Conservation analysis UCSC Genome Browser	DynaMut2 (ΔΔG Stability)	Allele frequency (GnomAD v.2.1.1)
rs757325337	c.59C>T	p.Ala20Val	S23-p by GSK3B	Reference = 0.818 Alternative = -0.034	Highly PhyloP: 1	-0.72 kcal/mol Destabilizing	0.00001426
rs1258632801	c.84G>T	p.Gln28His	S29-p, Y30-p and S33-p by GSK3B	Reference = -0.288 Alternative = -0.373	Moderately PhyloP: 0.812193	-0.13 kcal/mol Destabilizing	0.000003983
rs769203968	c.125C>T	p.Thr42Ile	T41-p and S45-p by GSK3B	Reference =-0.149 Alternative = -0.149	Highly PhyloP: 1	-0.31 kcal/mol Destabilizing	0.000007963
*rs1171472831	c.152A>G	p.Asn51Ser	S47-p	Reference = -0.544 Alternative = -0.544	Highly Phylo P: 0.928013	0.15 kcal/mol Stabilizing	0.000003981
rs1468458366	c.412A>G	p.Asn138Asp	Y142-p by FYN and PTK6	ND	Highly PhyloP: 0.92924	-0.35 kcal/mol Destabilizing	0.000003983
rs1172941347	c.569G>A	p.Arg190His	S191-p by CDK5	Reference = -1.739 Alternative = -1.739	Highly PhyloP: 1	-1.58 kcal/mol Destabilizing	0.000009295
rs147382769	c.583G>T	p.Val195Leu	S196-p	ND	Highly PhyloP: 1	-0.29 kcal/mol Destabilizing	0.000003989
*rs1242107231	c.983T>C	p.Met328Thr	Y331-p by PTK6 and Y333-p by SRC and PTK6	Reference = -2.282 Alternative = -0.442	Moderately PhyloP: 0.7968001	-1.94 kcal/mol Destabilizing	0.000003978
rs748148797	c.1660G>T	p.Gly554Cys	T556-p	ND	Highly PhyloP: 0.98938	-0.44 kcal/mol Destabilizing	0.000003979
rs754160678	c.2003A>G	p.Gln668Arg	Y670-p	ND	Highly PhyloP: 0.924743	-0.03 kcal/mol Destabilizing	0.000003980
rs772401455	c.2042C>T	p.Ser681Phe	S680-p and S681-p	ND	Moderately PhyloP: 0.78622	-0.66 kcal/mol Destabilizing	0.000003980
rs768012106	c.2149C>T	p.Arg717Cys	Y716-p	ND	Poorly PhyloP: 0.239134	-0.15 kcal/mol Destabilizing	0.000007084

* Variants with new post-transcriptional modification sites. VUS (Variant of Uncertain Significance), HGVS (Human Genome Variation Society), p=phosphorylation
ND (No Data).
